# Functional Polymers in Protein Detection Platforms: Optical, Electrochemical, Electrical, Mass-Sensitive, and Magnetic Biosensors

**DOI:** 10.3390/s110303327

**Published:** 2011-03-21

**Authors:** Jong-in Hahm

**Affiliations:** Department of Chemistry, Georgetown University, 37th & O Sts. NW., Washington, DC 20057, USA; E-Mail: jh583@georgetown.edu

**Keywords:** protein sensor, protein detection, polymer in sensor, sensor materials, nanotechnology

## Abstract

The rapidly growing field of proteomics and related applied sectors in the life sciences demands convenient methodologies for detecting and measuring the levels of specific proteins as well as for screening and analyzing for interacting protein systems. Materials utilized for such protein detection and measurement platforms should meet particular specifications which include ease-of-mass manufacture, biological stability, chemical functionality, cost effectiveness, and portability. Polymers can satisfy many of these requirements and are often considered as choice materials in various biological detection platforms. Therefore, tremendous research efforts have been made for developing new polymers both in macroscopic and nanoscopic length scales as well as applying existing polymeric materials for protein measurements. In this review article, both conventional and alternative techniques for protein detection are overviewed while focusing on the use of various polymeric materials in different protein sensing technologies. Among many available detection mechanisms, most common approaches such as optical, electrochemical, electrical, mass-sensitive, and magnetic methods are comprehensively discussed in this article. Desired properties of polymers exploited for each type of protein detection approach are summarized. Current challenges associated with the application of polymeric materials are examined in each protein detection category. Difficulties facing both quantitative and qualitative protein measurements are also identified. The latest efforts on the development and evaluation of nanoscale polymeric systems for improved protein detection are also discussed from the standpoint of quantitative and qualitative measurements. Finally, future research directions towards further advancements in the field are considered.

## 1. Introduction

The burgeoning area of proteomics has created an increasing demand for new materials which can effectively serve as active and/or passive components in various protein detection methods. Essential and crucial information such as evaluating protein levels, determining protein structure, assessing reaction dynamics and mechanisms of protein interactions, screening for the presence or absence of specific proteins, and analyzing protein activity can be obtained through protein detection methods. Therefore, improvements in current protein detection techniques are continuously being made in order to enable accurate and sensitive measurements. In addition, innovative detection systems involving novel materials are being developed to accomplish miniaturization, high throughput, and high sensitivity of protein assays. Such efforts come from many different research fields in the life sciences, physical sciences, engineering disciplines, as well as medical sciences. Possible applications of newly developed materials for improved protein detection can generally be placed into two categories. The first entails creating new materials for use as sensing (active) components, in which case specifically designed properties of the materials are actively involved in detecting signal production or transduction. Increasing sensitivity and selectivity of intended measurements are often the goal of research efforts related to this category. The second category pertains to the application of new materials as non-sensing (passive) components in protein measurements. Increasing biocompatibility and biostability, while concomitantly extending the lifetime of protein detection devices through resisting biofouling, are the goals of research endeavors in this category. For meeting such goals, crucial criteria for effective biodetection should be considered in the research and development process of new protein measurement systems, regardless of the material chosen and detection mechanisms. Ideal sensor materials should be easily and inexpensively produced in large quantities. They should also exhibit physical, optical, or electrical properties that improve the detected signal in comparison to those of conventional sensor materials. Ideal protein detection systems should not only facilitate qualitative but also quantitative measurements rapidly, accurately, and straightforwardly. They should be also capable of delivering efficient, parallel, and automated analyses that can be applied to large numbers of samples with reduced sample volume and reagent usage.

Polymers play an important role as one of the preferred materials to mediate various biologically essential constituents such as DNA, proteins, small molecules, and cells [1–7]. Polymers provide exquisite versatility and variety in chemical composition, allowing their physical, mechanical, and electrical properties to be tailored precisely during synthesis. Polymer can improve biosensor performance by conveniently incorporated into the fabrication process of nanobiosensor architecture [8]. Furthermore, their well-known and widely-available, surface chemistry of polymers can be exploited effectively for hosting an assortment of biomolecules. The biocompatibility and biodegradability observed in some polymers also make them very attractive for use in basic biological research and clinical diagnostic operation. Owing to these advantages, polymers are used extensively in a myriad of biomedical applications including genomics, proteomics, drug delivery, cell studies, and medical implants [1–7].

In order to assess the potential applicability of polymeric surfaces for a variety of analytical protein detection techniques, this review article first examines various existing methods for quantitative and qualitative measurements of proteins. The reviewed detection routes include optical, electrochemical, electrical, mass-sensitive, and magnetic approaches. The wide-ranging use of polymeric materials in these detection techniques is then overviewed and the desired properties of polymeric materials specifically required for protein detection are discussed. Current challenges are identified in the research and development area of various sensor platforms to investigate proteins. Difficulties facing both quantitative and qualitative protein measurements are also identified. Unique physical, chemical, and electrical properties of nanometer-sized polymeric materials are exploited to improve the sensitivity, selectivity, and throughput of protein measurements beyond the limits of current detection techniques. Such recent applications of nanoscale polymeric systems in various protein detection technologies are overviewed. In addition, latest efforts for developing quantitative nanoscale polymeric surfaces for improving protein detection are introduced. Areas of further investigation and development are contemplated for continued advancement of basic research and biotechnological applications involving proteins.

## 2. Existing Methods for Analytical Detection of Proteins

**Figure 1.**
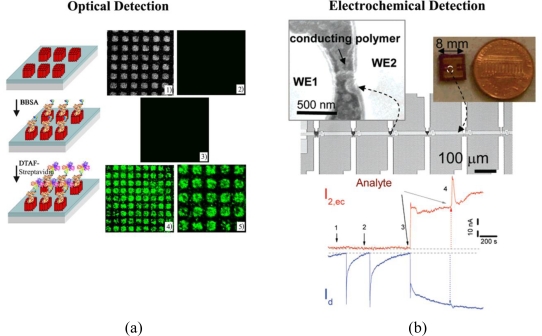

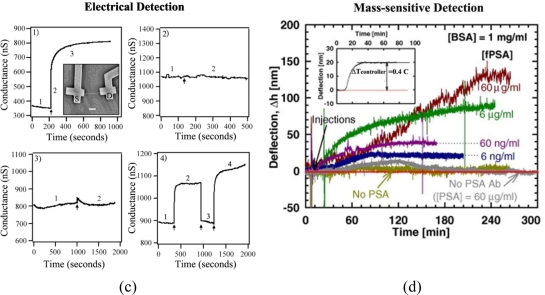
Different modes of protein sensors; (**a**) optical, (**b**) electrochemical, (**c**) electrical, and (**d**) mass-sensitive biodetectors. Adapted with permission from [9–12].

Quantitative and qualitative protein detection is currently achieved through various experimental means based on changes in optical, electrochemical, electrical, physical (mass-sensitive), and magnetic signal, see examples in Figure 1. Table 1 lists commonly employed experimental methods for analytical protein detection in each category.

**Optical Methods.** Optical detection is the most widely used mechanism and serves as the basis for many beneficial techniques such as colorimetry, ultraviolet-visible (UV-vis) spectrophotometry, and fluorometry. Colorimetric techniques such as the Bradford assay [13], the Lowry assay [14], the biuret assay, and the bicinchoninic assay [15] are also commonly used due to their ease of performance and low-cost [16]. UV- and VIS-absorption spectroscopy techniques are used in stand-alone assays or in conjunction with dye-based or other chemical-based assays, and are typically the preferred method for obtaining quantitative data monitored in real time [17]. Dyes and chemicals used in these assays typically contain Coomassie Brilliant Blue G-250 in the Bradford assay, Cu^2+^ ion along with Folin and Ciocalteu’s enhancing reagent in Lowry and biuret assays, and Cu^2+^ ion with bicinchoninic acid reagent in the bicinchoninic assay. However, these dyes and chemicals can suffer from interferences of other common reagents in assays such as acids/bases, buffers/salts, and detergents. This propensity can, in turn, decrease the protein detection sensitivity of the techniques. In order to reduce these problems, the intense surface plasmon bands of inert metallic nanoparticles have been recently employed as the source of colorimetric enhancement [18]. Another method is derived from scanometric readout of the optical signal of metallic nanoparticles as signal enhancement basis and this approach reports higher detection sensitivity than conventional colorimetric techniques [19,20].

Methods based on fluorescence are especially popular and prevalent both in laboratory and clinical applications due to high sensitivity and flexibility in their operating modes. When compared to the colorimetric techniques, whose protein detection capability is in the range of a few to hundreds of micrograms, fluorescent dye-based approaches involving the use of o-phthalaldehyde [21], Fluorescamine [22], or 3-(4-carboxybenzoyl)quinoline-2-carboxyaldehyde [23,24] in assays can improve the protein sensitivity to tens of picograms. Another advantage in using fluorescence methods is the availability of a wide variety of fluorophores and their relatively stable performance in various assays. More recently, various sophisticated analytical techniques have been developed not only to quantify the amount of particular proteins but also to analyze interactions between multiple proteins. Examples of these analytical techniques include fluorescence resonance energy transfer (FRET) [25], fluorescence liftetime imaging microscopy (FLIM) [26–28], fluorescence correlation spectroscopy (FCS), and fluorescence recovery after photobleaching (FRAP). FRET is used for important applications such as determining localized structures of interacting proteins, protein folding behavior, conformational dynamics, and reaction mechanisms [29–31]. FRAP and FCS are exploited to gather information on protein binding kinetics and protein complex formation [32,33]. These techniques provide access to a multitude of measurement parameters such as local concentration, reaction kinetics, structural conformation, and dynamic interaction behavior of proteins. The detection resolution of these techniques can be as high as a single molecule as demonstrated by single molecule FRET experiments [34]. However, some of these fluorescence-based methods can be prone to high background noise during optical measurements and may present difficulties in quantitative data analysis.

The aforementioned optical techniques often require the use of labels such as fluorophores (organic dyes, semiconductor nanocrystals, and quantum dots), other inorganic chemical reagents, and biological tags (enzymes with chromogenic agents, native and modified green fluorescent proteins). More recently, surface plasmon resonance (SPR)-based methods based on the change in refractive index have been applied for time-resolved investigation of proteins without the need for dyes or chromogenic agents [35–38]. SPR offers the benefit of label-free detection and high sensitivity for single analytes. However, this technique currently experiences limitations in sample types and multiplexing capability. SPR cannot be effectively applied to measure low concentrations of proteins with low molecular weight since the present application requires greater than 1 pg/mm^2^ of protein coverage. Despite efforts to develop multichannel SPR sensors [39], the technique cannot efficiently handle a large number of protein samples simultaneously in a rapid manner.

**Electrochemical Methods.** Electrochemical approaches are useful for detecting protein samples as they have the important advantage of offering label-free and quantitative measurement capability [40–42]. The majority of optical detection methodologies is faced with the significant challenge of inferring accurate and quantitative results from the collected optical signals during measurements. In contrast, electrical detection methods are quantitative and more readily amenable to direct interpretation of data. Electrochemical measurements are typically carried out without the use of any labels and the signal is read directly from the protein samples. Combined with their potential as miniaturized, lab-on-a-chip devices for use in point-of-care measurements, electrochemical methods can serve as an alternative means for protein detection to radioactive-, fluorescence- and enzyme-based assays [41–45]. Common operating types of electrochemical detection are potentiometric, capacitive, and amperometric modes where the most common type is amperometric transducers. The detection sensitivity of these techniques is in general not as high as that of fluorescence-based methods. In an attempt to improve the detection limit, indirect electrochemical detection is performed using an auxiliary reaction which involves a labeling (redox active) compound for signal generation. This approach is also helpful for detecting proteins that are not electrochemically active within the commonly applied potential range. However, such methods face difficulties in additional label usage and signal interpretation, similar to those in many optical techniques. Besides sensitivity, other challenges associated with electrochemical detection are issues related to electrode fouling, electrochemical stability of reagents, and side electrochemical reactions. New materials such as gold nanoparticles, carbon nanofibers, carbon nanotubes and tin oxide are employed as electrodes in the recent applications to reduce electrode fouling while increasing sensitivity [46–51].

**Electrical Methods.** Electrical detection operated by one- and two-dimensional field effect transistor (FET) devices is also applied for label-free, real-time monitoring of protein systems [52–55]. These devices are conventionally fabricated from silicon using a top-down approach for planar FETs and from nanomaterials such as carbon nanotubes and silicon nanowires using a bottom-up approach for one-dimensional FETs [56–58]. Electrical detection strategies, especially one-dimensional FETs, offer a promising potential for integration into small detection devices in an array geometry [59]. Research efforts are continuously being made to assemble sensing materials in a periodic arrayed fashion to facilitate device integration and manipulation [53]. They can rely on the fabrication practices formerly established by the silicon industry and also on recent advances in nanomaterial processing. However, these techniques are considered to be in their very early stages of development. They are not yet commonly employed in the laboratory or clinical research environments as the previously discussed methods.

**Mass-Sensitive Methods.** Mass spectrometry (MS) is one of the central analytical techniques in proteomics [60–64]. It has been extensively employed as an operating principle for various protein detection methods such as time-of-flight secondary ion mass spectroscopy (TOF-SIMS) and nanostructure-initiator mass spectrometry (NIMS) [61,65,66]. These methodologies are effective for obtaining kinetic and activity information of proteins. MS based methods are useful for detecting peptides and small proteins (up to the mass-to-charge ratio, m/z, of 25 kDa approximately) but not as successful for measuring larger proteins. This drawback severely constraints the application of this beneficial technology since many proteins, including cytokines, growth factors, enzymes, and receptors have molecular weights exceeding 25 kDa. The microchannel plate detector, typically used in MS for protein imaging, is not well suited for detecting high m/z ions and is prone to detector saturation and signal suppression when analyzing complex mixtures. The extensive processes required for sample preparation such as extensive purification, stable isotope labeling, and chemical tagging can complicate the measurement and decrease the detection efficiency. Therefore, the overall utility of the MS-based methods suffers from varying degrees of difficulties in these aspects. Other mass-sensitive techniques include quartz crystal microbalance (QCM) and microcantilevers. They are employed to detect proteins by monitoring and recording changes in resonance frequency, beam deflection, or electrical resistance of a sensor resulted from the mass of bound proteins on the sensor surface [67–70]. Quartz crystal resonators and piezoresistive cantilevers are used as sensing materials for the QCM and microcantilever technique, respectively. In order to increase the sensitivity of traditional QCM devices, platforms featuring a high frequency QCM (greater than 10 MHz resonance frequency in liquid) and a thin resonator (several micrometers in thickness) are considered [9,71,72]. Compared to the relatively frequently used QCM techniques, microcantilever-based protein detection is still in its exploratory stage and its application is currently limited to a pilot-stage and used for laboratory scale detection.

**Magnetic Methods.** Magnetic detection routes involving the use of magnetic particles are also employed for protein analysis. Nuclear magnetic resonance (NMR) spectroscopy has been conventionally used to probe protein structures and interactions at atomic resolution [73–76]. The major limitations of this technique in protein detection are low sensitivity, size range of detectable samples, and the need for isotopic labels. In order to increase sensitivity, high field magnets and cryogenically cooled probes have been used in the measurements. Multidimensional NMR (2D–4D) as well as uniform isotopic labeling strategies has widened the range of size and complexity of protein samples that can be measured by NMR. Magnetic protein detection is applied for diagnostic and treatment purposes as well. Examples include diagnostic magnetic resonance, magnetoresistive sensor, and chip-based nuclear magnetic resonance. These approaches offer more benefits for protein detection at the systems level than on the molecular level [77–79]. The necessities for such measurements, such as the employment of labels with specific magnetic properties and complicated instrumentational requirements for detection, restrict their routine application in the basic laboratory setting, especially for investigating isolated proteins at the molecular level. Rather, magnetic methods are commonly incorporated into other protein detection techniques as an upstream process to facilitate separation and collection of target proteins before measurements.

## 3. Polymers Currently Used in Analytical Detection of Proteins

The versatile and beneficial properties of polymers have been exploited to facilitate protein detection in all areas of the aforementioned methods. A wide range of polymeric materials is already commercially available or synthetically accessible. Their surface chemistry can be easily tailored for the immobilization of proteins. In addition to these benefits, cost-effective polymers can be produced in large quantities. They can be easily handled and fabricated into assorted platforms. Polymers can withstand the thermal fluctuations that are typically required in standard biomedical protocols. Owing to these advantages, polymers have been used not only as sensing materials and signal-enhancing components of various detection devices but also as supporting substrates and mediating layers for promoting/suppressing protein adsorption. As a result, polymers are commonly found in protein detection technology, especially based on optical, electrochemical, and mass-sensitive mechanisms.

**Figure 2.**
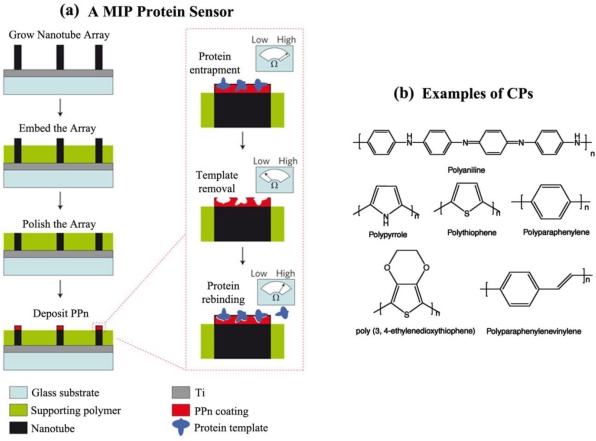
Molecular imprinting polymers (MIPs) and conducting polymers (CPs). (**a**) An electrochemical protein sensor employing a MIP and (**b**) typical examples of CPs. Adapted with permission from [80] and [81].

When choosing polymers for protein detection, multiple factors such as biocompatibility and hydrophobicity/hydrophilicity are carefully considered for the specific need in each application. Two main reasons for applying polymers in various protein detection systems are to increase specificity and sensitivity. For example, some polymers [82–84] have been frequently employed to provide an additional layer for promoting protein adsorption and for increasing protein stability on various sensor surfaces. On the other hand, other polymers have been used as inhibition layers to suppress nonspecific protein adsorption on sensors [85–87]. Molecular imprinting polymers (MIPs) are applied to the electrochemical detection of proteins in order to increase biomolecular selectivity. They permit the creation of specific protein recognition sites in synthetic polymers through template molecule-assisted copolymerization of functional monomers. When the template molecules are removed from the polymer, complementary binding sites to subsequent template molecules are constructed in the polymer [88,89]. Figure 2(a) is one such example of MIP protein sensors. Conducting polymers (CPs), a group of polymers exhibiting good electrical conductivity (10^−11^ to 10^3^ S/cm with a carrier concentration ranging between 10^12^ and 10^19^/cm^3^), have been used to increase the detection sensitivity of protein detection systems such as FETs [11,81,90,91]. Figure 2(b) displays typical examples of CPs. The following section overviews various use of polymeric materials in each area of protein detection, with an emphasis on their role to increase selectivity and sensitivity of desired measurements. Table 2 classifies various polymers used in protein detection according to their assorted properties and relevance in each detection category.

**In Optical Detection.** Currently, optical detection methods are predominantly employed in basic biological and biomedical research as well as in biotechnology applications. A large number of such optical protein assays involves polymeric supports as detection platforms. Therefore, the role of polymers in this detection area is becoming increasingly important and their application is rapidly expanding. Before their use, several key characteristics of a polymer are carefully assessed before its use. Examples of evaluated criteria for a candidate polymeric material may include biocompatibility and biostability. They are also screened for the physical, biological, and optical properties typically required for protein measurements. Commonly and widely-used fluorescence-based detection requires particular optical properties from candidate polymeric materials including low intrinsic fluorescence background and high optical transparency to the excitation and emission wavelengths which are typically used in fluorescence measurements.

Despite the wide availability of polymers in general, these requirements restrict their application in biodetection to a smaller subset of polymers demonstrating the desired characteristics. For example, polycarbonate (PC), polyurethane (PU), polydimethylsiloxane (PDMS), polystyrene (PS), polymethylmethacrylate (PMMA), cycloolefin-based polymers, and combinations of these polymeric materials have been used to a great extent in the past. Modifications to the physical and chemical structures of these workhorse polymers have expanded their use to a certain degree. In addition to the macroscopic scale polymers produced via thermal and injection molding, polymeric nanofibers generated via an electrospinning process have been utilized in protein assays to provide an increased surface area for protein assembly [92,93]. The chemical heterogeneity of polymers employed in protein measurements has also been altered by several means in order to localize proteins to certain areas and to incorporate specific chemical properties into these areas. Some of the applications of electrospun nanofibers include polymeric mixtures and copolymers instead of homopolymers [86,94,95]. Recently, ultrathin films of polymeric blends and diblock copolymers have been utilized as protein substrates for high-density optical detection [96–102].

Various fabrication techniques are used during the manufacturing process of microwell plates and protein arrays in order to incorporate proteins effectively onto polymeric surfaces. These techniques include imprint lithography [103–105], molecular imprinting [80,88,89], microcontact printing [106–108], photolithography [109,110], and dip-pen lithography [111,112]. Polymers are employed extensively to carry out these techniques effectively. PDMS and PMMA are used as stamp materials for microcontact-printing proteins to various substrates [113,114]. PS, polyacrylic acid, polyethylene glycol (PEG), polyvinyl alcohol, as well as photo-reactive polymers are often involved in the spotting and casting process of proteins via photoimmobilization [109,110]. On the other hand, materials such as PDMS, polyphenol, polypyrrole, photoresist polymers, and molecular imprinting polymers are employed in producing a protein detection platform with a molecular recognition capability [80,115,116].

**In Electrochemical Detection.** Polymers are applied frequently to coat a sensor electrode in an electrochemical setup to increase the detection sensitivity of the device. PEG has been utilized to increase antibody adsorption and to provide stable antibody-binding sites on an electrode surface [117]. A poly(pyrrole-N-hydroxysuccinimide) film on an impedimetric electrode has been used to increase the amount of immobilized antibodies [118]. A thermoresponsive polymer, poly-N-isopropylacrylamide-ferrocene, has been utilized for sensitive electrochemical detection of glucose dehydrogenase [119]. Polyphenol has been employed as a surface receptor layer in a carbon nanotube-based electrochemical impedance sensor, resulting in increased detection sensitivity of a device with molecular imprinting capability [80]. In other cases, polymers are utilized to establish new chemical, biological, and electrical functionalities of sensor electrodes. Electropolymerized composites of polypyrrole, polypyrrolepropylic acid, and Au nanoparticles have been used in an impedimetric sensor in order to provide hydrophilicy, electroactivity, and electrical conductivity, respectively [120]. Nanostructured polyaniline film has been applied onto indium tin oxide glass electrodes for capacitive protein detection to increase selectivity by covalently linking target antibodies onto the sensor surface [121]. Chitosan fiber coating is used on a gold wire to couple the easy biofunctionalization property of the polymer with the signal transduction capability of a conducting wire [122]. Highly charged polymers such as poly-l-lysine have been used to modulate the rectification properties of nanopipette electrodes [123]. In some cases, polymers have been used as flexible supporting substrates to accommodate printed electrode devices on their surfaces. Poly(ethylene terephthalate) is used to construct single-walled carbon nanotube-based, electrochemical glucose sensors [124]. The same polymer has been also used as a membrane material to house conical gold nanotube sensing elements in nanopore resistive-pulse detection [125].

**In Electrical Detection.** Similar to their use in electrochemical detection, one type of polymeric application in FET devices is limited to non-participating components in electrical detection. Polymers have been used for increased sensitivity and specificity by discouraging specific binding of target molecules to the non-sensing region and through reducing device contamination from random protein adsorption. Polymers are also used to protect the vital and sensitive electrical components of FET devices for durable and repeated measurements. For example, electropolymerized pyrrole propylic acid served as a protective layer on the non-sensing components in a TiO_2_ nanowire FET [126].

In other cases, the unique electrical properties of some polymers were exploited as acting sensors of semiconducting channels in FET sensors. Conducting polymers have demonstrated their utility as a biosensor material in these applications, although their current utility is limited due to difficulties in device integration and manufacturing using traditional microfabrication processes [11,81,90,91].

**In Mass-Sensitive and Magnetic Detection.** In mass-sensitive detection, the use of polymers is focused on promoting selective binding of target proteins and their stability upon protein adsorption, while decreasing random protein binding to a sensor surface. Hydroxyethyl- and ethyl(hydroxyethyl) cellulose as well as hydrophobically modified analogues of these polymers were demonstrated to make the surface of Au, a common QCM sensor material, partially protein-repellent or completely free of biofouling [127]. Phospholipid (2-methacryloyloxyethyl phosphorylcholine) polymer has been used in QCM as an antibody stabilizing agent whose role is to reduce nonspecific binding of an antigen solution and to suppress denaturation of immobilized antibodies on a QCM sensor [128]. Molecularly imprinted polymers are also employed in QCM to increase selectivity of protein detection [129]. In addition, a photoderivatized method was demonstrated through the insertion of photoprobes on PEG-coated sensor surface to increase selective binding of proteins [130].

In magnetic assays typically involving magnetic nanoparticles or beads, the application of polymers are generally limited to device substrates and nanoparticle coatings. Thermoplastic materials can function as cost-effective and versatile alternatives to traditional silicon- or glass-based substrates in biodetection for rapid prototyping and industrial scale fabrication of sensor devices. Cyclic olefin copolymers are used as substrates for a lab-on-a-chip platform for magnetic bead-based immunoassay with fully on-chip sampling and detection capabilities [131]. Polymers can serve as encapsulating layers for the magnetic particles to prolong their stability, enhance their chemical functionality, and prevent them from aggregating with one another. Multifunctional copolymers are often chosen for these purposes. Examples include an amphiphilic triblock copolymer of methoxy-PEG-poly(glutamate hydrozone doxorubicin)-poly(ethylene glycol)-acrylate, a micellar copolymer of PEG-poly(β-amino ester)/(amido amine), and a copolymer of styrene and glycidyl methacrylate [132–134].

**Figure 3.**
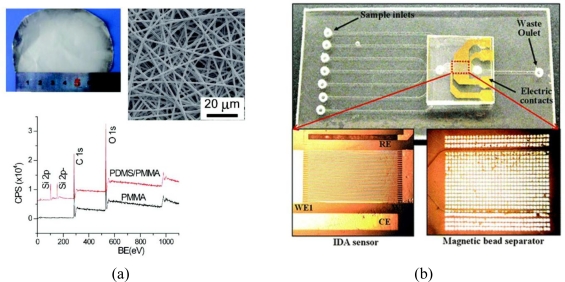
More examples of functional polymers; (**a**) electrospun polydimethylsiloxane (PDMS)/polymethylmethacrylate (PMMA) nanofibers and (**b**) an integrated immunoassay device on cyclic olefin copolymer. Adapted with permission from [86] and [131].

Figure 3 shows (a) an electrospun nanofiber sensor consisting of PDMS/PMMA as described in the optical detection section and (b) an interdigitated array (IDA) for magnetic bead-based immunoassays fabricated into a lab-on-a-chip device on cyclic olefin copolymer.

## 4. Nanoscale Polymeric Surfaces for Enhanced Protein Assembly

Advances in nanoscience can be exploited in protein assembly to achieve an additional degree of control in density and payload of surface-bound proteins for improved analytical measurements. Although the majority of current polymer applications in protein arrays involves chemically homogeneous materials prepared on a macroscopic level, the chemical complexity and length scale of proteins are in better agreement with chemically heterogeneous, nanoscale polymeric surfaces. Several recent approaches investigated potential applications of self-assembled diblock copolymer nanodomains that exhibit periodically varying chemical compositions.

For example, the unique phase separation behavior of a block copolymer, polystyrene-*block*-polymethylmethacrylate (PS-*b*-PMMA), has been shown previously to expose both block components to the air/polymer interface under carefully balanced thermodynamic conditions [135]. This phenomenon generates spatially periodic, self-assembled, nanoscale polymeric domains consisting of the different chemical constituents of the two polymeric components, whose scale and geometry reflect the chemical and physical properties of the polymer [136–138]. Their phase diagram dictates the packing nature and orientation of the resulting polymer chains whereby their microphase separation behavior is predictable based on a mean field theory [139–141]. Therefore, the repeat spacing and surface geometry of the diblock copolymer can be controlled by changing the molecular weight and compositions of the two blocks.

Another category of amphiphilic diblock copolymers exhibits micellar assembly above a critical polymer concentration. Their fascinating micellar properties and dependence on diblock copolymer characteristics are extensively studied for polystyrene-*b*-polyacrylic acid, poly(ethylene-propylene)-*b*-polyethylene oxide, polystyrene-*b*-poly(2-vinylpyridine) and polystyrene-*b*-poly(4-vinylpyridine) [142–145]. The exact structures and configurations of the resulting micelles or aggregates are determined by the composition of the diblock polymer, the length of each polymer segment, the polarity of the solvent, and the relative solubility of each polymer block in the solvent. These chemically alternating and self-assembling polymeric domains can serve as convenient self-constructed templates for nanoscale arrangement of the desired biocomponents.

**Figure 4.**
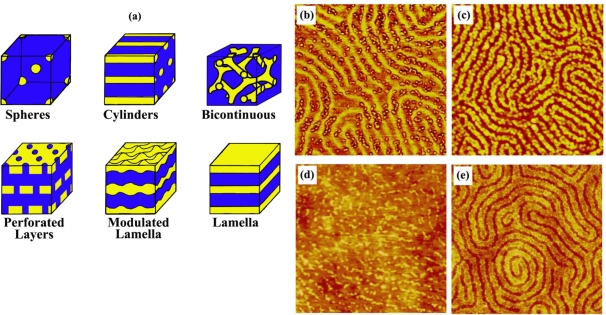
One-dimensional diblock copolymer templates of PS-*b*-PMMA and protein assembly behavior on them; (**a**) various nanoscale templates resulting from phase-separated nanodomains of diblock copolymers, (**b** and **c**) immunoglobulin G molecules assembled on PS-*b*-PMMA, and (**d** and **e**) protein G molecules on the same template. Panels (b) through (e) are 1 × 1 μm atomic force microscopy (AFM) images. Adapted with permission from [97].

**Figure 5.**
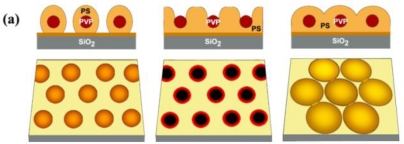

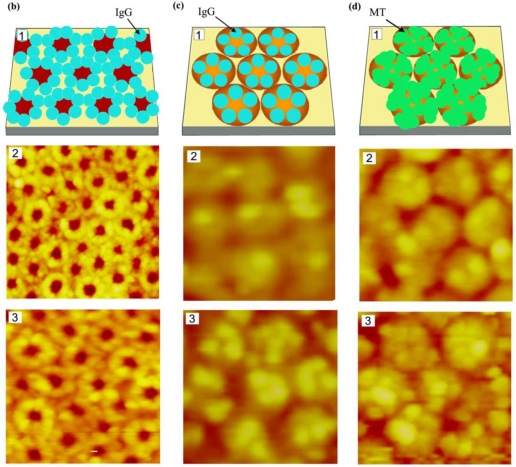
Two-dimensional diblock copolymer templates of PS-*b*-PVP and protein assembly behavior observed on them; (**a**) various nanoscale templates resulting from chemical modification of nanodomains in micellar-forming diblock copolymers, (**b** and **c**) immunoglobulin G molecules on (b) open and (c) reverted PS-*b*-PVP templates, and (**d**) mushroom tyrosinase molecules assembled on a reverted PS-*b*-PVP template. The atomic force microscopy (AFM) scan size in panels (b) through (d) corresponds to (b): (2) 300 × 300 nm, (3) 180 × 180 nm, and (c and d): (2) 300 × 300 nm, (3) 180 × 180 nm. Adapted with permission from [100].

Recently, preferential interaction of several model proteins with PS and their selective segregation on the PS regions were monitored on the surface of phase-separated, PS-*b*-PMMA diblock copolymer ultrathin films [97,99]. In addition to these methods for arranging proteins with one-dimensional control over repeat spacing, spatial control over two dimensions was accomplished by using micelle-forming diblock copolymers. Polystyrene-*b*-poly(4-vinylpyridine) (PS-b-PVP) was effectively used for the self-assembly of surface-bound, two-dimensional, nanoscale protein arrays [100]. A straightforward method to produce protein patterns of different geometries and sizes study was also established in the same study by manipulating topological structures of the underlying PS-*b*-PVP templates via various chemical treatments. Figures 4 and 5 display various nanodomain templates in diblock copolymers and the characteristic protein assembly behavior on such templates of PS-*b*-PMMA and PS-*b*-PVP ultrathin films, respectively.

## 5. Application of Nanoscale Polymers in Improved Analytical Protein Measurement

Several nanoscale polymeric systems are assessed for their potential to improve analytical protein detection through various means. For instance, highly dense protein nanoarrays with specifically tailored local functionality can be beneficial for smart monitoring and diagnosis of protein markers. The employment of nanoscale polymeric motifs can greatly facilitate protein detection due to their increased surface-to-volume ratio and unique properties occurring at the nanoscale, especially in detection environments where polymers participate actively in the detection as sensing components. However, only limited application of nanoscale polymeric systems to such function has been demonstrated so far. These latest approaches, representing the application of nanoscale polymers for improved protein detection as well as experimental techniques for generating nanoscale polymeric features, are discussed below for each detection mechanism.

In optical detection involving fluorescence and visible light, various model proteins were self-assembled onto nanoscale domains of PS-*b*-PMMA diblock copolymer ultrathin films for both qualitative and quantitative analysis. They include horseradish peroxidase, mushroom tyrosinase, enhanced green fluorescent protein, bovine immunoglobulin G, bovine serum albumin, and protein G. When the activity and stability of these common and useful proteins were assessed, PS-*b*-PMMA-bound proteins retained approximately 85% of their free activity after surface adsorption [101]. On the other hand, protein molecules bound on the hexagonally-packed PS-*b*-PVP nanodomains retained 78% of their activity when measured in solution [98]. Such quantitative analysis was possible due to the use of well-defined nanoscale polymeric templates as well as the combined measurement techniques of atomic force microscopy (AFM) and UV-vis spectroscopy. The number of surface-bound protein molecules is determined by the size of the nanoscale polymeric domain as well as that of the protein, which can be predicted and confirmed by AFM imaging. UV-vis analysis can then be carried out for bioactivity measurements for a known number of proteins in two different environments. One environment contains proteins bound on polymeric surfaces and the other involves the same number of proteins moving about freely in solution. Protein functionality in this approach can be quantitatively compared between the two cases.

**Figure 6.**
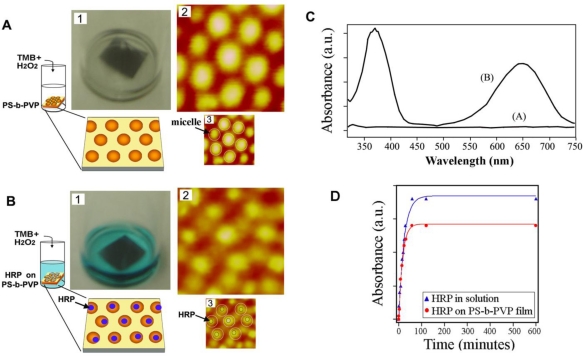
Quantitative and qualitative HRP activity measurements on PS-*b*-PVP nanodomains. (**A**) Control experiment without HRP molecules on PS-*b*-PVP. (**B**) (1) Assay carried out with HRP molecules on PS-*b*-PVP. AFM panels in (A) and (B) are 180 × 180 nm in scan size. (**C**) (A) No absorbance peaks from the control experiment involving only the PS-*b*-PVP template. (B) Characteristic UV/VIS absorbance peaks were monitored due to HRP bound on PS-*b*-PVP. (**D**). UV/VIS absorbance of HRP molecules monitored over time in solution (data shown in blue) and on PS-*b*-PVP micelles (data shown in red). When compared to the activity of HRP molecules in solution, HRP molecules bound on PS-*b*-PVP showed 78% of the activity. Adapted with permission from [100].

Figure 6 displays both quantitative and qualitative data of HRP activity measured and compared between PS-*b*-PVP bound state *versus* free state. Although not yet demonstrated, diblock copolymer-guided methods of protein assembly have the potential to be used effectively outside the optical detection setting. For example, the aforementioned protein assembly on nanoscale polymeric templates can be applied to QCM and SPR sensor surfaces for improving the detection sensitivity beyond the current capabilities of macroscopic scale polymeric systems in those sensors.

In electrochemical and electrical detection, nanoscale CPs such as polypyrrole, poly(pyrrolepropylic acid), and poly(3,4-ethylenedioxythiophene) nanowires have been used as semiconducting channels in a FET type of devices [146–151]. Electronic conduction in these polymeric nanowires depends on the width of the nanowire channel and occurs through bulk conduction due to their high density of electronic states. Therefore, their diameter-dependent property in electrical conductivity is exploited to improve tunable sensitivity of protein measurements.

Both in optical and mass-sensitive detection, the use of nanoscale polymeric templates has been demonstrated in the preparation of sensor platforms in order to increase the amount of proteins on sensor surfaces. Nanoscale polymeric templates in these cases were obtained through various printing and lithographic methods. An electron beam and a scanning probe microscope were used to create nanoscale polymeric patterns on sensor platforms both physically and chemically. Sensor surfaces were modified by using an electron beam to inscribe topological polymeric patterns for subsequent protein binding, and in some cases, selective sites of polymeric surfaces were chemically activated for subsequent protein attachment or resistance [152–155]. Nanoimprint- and nano-lithography have been used to deliver nanoscale polymeric patterns on sensor surfaces upon contact with a silicon mould [104,156,157]. Nanoscale features on the mould piece were often defined by electron beam writing before its application. An electron beam, instead of light, was also used to crosslink pre-conjugated monomers on sensor surfaces to generate nanoscale polymeric patterns [158,159]. In addition, dip-pen lithography and other scanning probe tip-based methods have been exploited to draw nanometer scale polymeric features on substrate surfaces [160].

## 6. Current Challenges and Areas of Improvements

Regardless of the specific roles that polymers play in each type of protein detection method, an ideal application of polymers should promote not only qualitative but also quantitative detection both rapidly and cost-effectively along with high specificity and sensitivity. The chemical complexity and heterogeneity of protein analytes need to be addressed when selecting appropriate polymeric surfaces in the detection scheme in order to maintain the natural conformation and activity of proteins during intended measurements. Quantification of protein molecules via signal interpretation should ideally be direct and straightforward. These important characteristics need to be considered for all modes of polymeric applications in protein detection, whether they are used as substrate platforms in optical arrays, incorporated into the electrodes in electrochemical cells, coated onto the sensor layers in mass-sensitive resonators, or applied to the non-sensing regions of FET devices. In addition to the relatively small subset of polymers that are used currently in protein detection, more biocompatible and biostable polymeric materials should be developed and assessed for their effectiveness in a protein detection setting.

In optical detection involving an array-style of protein screening, ideal polymeric substrates should enable quantifiable, parallel, small-volume assays to be readily applied to large numbers of samples. They should also feature reliable placement of protein molecules in a well-defined, highly dense pattern. Current difficulties associated with the application of proteins printed on polymeric surfaces lie in multiple areas of sensor development and detection. They include the precise control over protein density, spot density, protein orientation, spotting uniformity, array standardization, array stability, and detection sensitivity. Increasing the signal-to-noise ratio of current optical detection platforms is also of great significance, especially when the detection is carried out without any means of signal amplification. In addition to the need for an increased signal-to-noise ratio in many fluorescence-based techniques, correlating optical signal intensity to protein concentration reliably and accurately is a demanding task in these techniques. More standardized and direct methods to compare the measured optical signal to the amount of proteins in the reaction are currently warranted. Improvements in protein spotting processes are necessary in order to produce uniformly printed proteins on various optical detection platforms. Another important challenge in the quantification of conventional assays, in which the exact number of biologically functional biomolecules participating in reactions can be easily and meaningfully compared between assays, still needs to be addressed effectively. Advanced protein printing and assembly methods, capable of producing a well-defined number of proteins on polymeric surfaces that are consistently distributed on each spot in the array, are highly needed.

In electrochemical and electrical detection, quantitative analysis of the detection signal is more straightforward than that in optical methods. Measured signal of current (or impedance) and conductance (or resistance) can be directly correlated to the amount of proteins contributing to the bioreaction. However, challenges may still arise as a result of the innate properties of proteins. Proteins are structurally and chemically complex, often requiring specific chemical and biological environments to remain active. Different proteins can show vastly dissimilar chemical properties and, thus, a single standardized condition cannot be simultaneously applied for manipulating a large number of proteins. Yet, factors such as the degree of protein denaturation and the orientation of properly aligned proteins on polymeric surfaces can significantly influence the detection results, especially when the polymers act as a part of the sensing regions of the device. Proteins on sensor surfaces may not be properly detected in all modes of protein detection, particularly in the cases of denaturation on polymeric surfaces leading to the loss of spectroscopic signature (in optical detection), redox activity (in electrochemical detection), surface charges (in electrical detection), and physical integrity (in mass-sensitive detection). Even for appropriately folded proteins, measurement techniques can detect the analytes only when they present functional subunits or binding pockets along the direction of their subsequent interaction. Accurate assessment of proteins on sensor surfaces participating actively in the detection is, therefore, critical especially for meaningful and quantitative protein measurements.

Proteins tend to stick to many surfaces indiscriminately when their assembly is not carefully controlled. Therefore, in many operating modes of protein detection, effective passivation of certain surface areas often becomes a necessity in order to avoid cross and carry-over contamination and to increase the signal-to-noise ratio. In detection schemes using proteins assembled on diblock copolymer nanoscale domains, self-passivation is intrinsically achieved through the self-selective nature of proteins to a preferred domain in the chemically heterogeneous polymeric templates. When compared to the conventional, chemically homogeneous substrates, this phenomenon offers a distinct advantage over the approach involving self-assembling chemically-heterogeneous polymeric templates. However, topological defects in the phase-separated diblock copolymer templates can limit the effectiveness of the subsequent protein adsorption and, thus, can affect protein measurements. Research efforts have been made in the past to identify, understand, and control surface defects such as disclinations and dislocations during the thermal annealing process of the polymeric surfaces above their glass transition point [136,138]. In addition to the thermal annealing control, external measures such as an electric field [161], shear field [162], controlled solvent evaporation [163], annuli formation [137], as well as other geometric and chemical constraints [164–166] were used to induce long-range alignment of polymeric nanodomains. In order to broaden the applicability of these nanoscale diblock copolymer systems in protein detection, more effective and convenient methods to produce defect-free nanodomains with correlation lengths spanning macroscopic dimensions need to be yet developed.

## 7. Concluding Remarks

Accurate detection of proteins is extremely critical in many important areas of biological and biomedical research. Valuable information such as evaluating protein levels, determining protein structures, assessing reaction dynamics and mechanisms of protein interactions, screening for the presence or absence of specific proteins, and analyzing protein activity can be obtained through protein detection. Considerable research efforts are therefore underway for improving existing methodologies and techniques for the commonly used optical, electrochemical, electrical, mass-sensitive, and magnetic detection. In addition, nanoscale polymeric materials are assessed for their potential for better protein detection and employment in novel detection systems. This article reviews such efforts, especially focusing on the use of macro- and nano-scale polymeric materials to improve sensitivity, selectivity and analytical ability of protein measurements. Both qualitative and quantitative approaches for protein measurements are discussed in this article. Challenges involved with protein detection in general as well as specific difficulties associated with each detection technique are identified. Current and anticipated hurdles for using macroscopic and nanoscale polymeric materials in protein detection are also discussed. Finally, future research areas pertinent to alleviating and potentially overcoming the identified drawbacks in protein detection are contemplated.

## Figures and Tables

**Table 1.** Various protein detection sensors involving polymers; optical, electrochemical, electrical, mass-sensitive, and magnetic modes.

**Detection Mechanism**	**Detection technique**		**Detection Signal**
Optical [8–11], [16–19], [30–34]	Colorimetric assay	Bradford, Lowry	Color
		Biuret	
		Bicinchoninic	
	UV-Vis absorption spectroscopy	Enzyme-linked immunosorbent assay	Absorption maximum of a chromogenic agent
	Fluorescence imaging/spectroscopy	Fluorescence imaging	Fluorescence emission
		Fluorescence resonance energy transfer	
		Fluorescence liftetime imaging microscopy	
		Fluorescence correlation spectroscopy	
		Fluorescence recovery after photobleaching	
	Surface plasmon resonance spectroscopy		Refractive index
Electro-chemical [35–40]	Potentiometric		Voltage
	Capacitive		Capacitance
	Amperometric		Current
Electrical [47–54]	Field effect transistors	One-dimensional	Conductance/Current
		Two-dimensional	
Mass-sensitive [55–68]	Mass spectroscopy	Mass spectrometry	Molecular weight
		Time-of-flight secondary ion mass spectroscopy	
		Nanostructure-initiator mass spectrometry	
	Quartz crystal microbalance		Resonant frequency
	Microcantilevers		
Magnetic [69–75]	Nuclear magnetic resonance spectroscopy		Chemical shift

**Table 2.** Properties of polymers exploited in different types of protein sensors as well as sensor regions of applied polymers.

**Properties of polymers**	**Types of polymers**	**Applied Detection Category**	**Applied Sensing Area**
Physical	Macro or larger size (Polymers produced via thermal/injection moulding Electrospun fiber bundles)	Optical, Electrochemical, Electrical, Mass-sensitive, Magnetic	Active Passive
	Nanosize (Phase separated nanodomains in block copolymers, Electrospun nanofibers, Polymeric nanowires)	Optical, Electrochemical, Electrical, Mass-sensitive, Magnetic	
Chemical	Single component (Homopolymers)	Optical, Electrochemical, Electrical, Mass-sensitive, Magnetic	Active Passive
	Multiple components (Linear or branched copolymers, Polymer mixtures/blends, Amphiphilic polymers)		
	Molecular imprinting polymers (Polymer with built-in molecular recognition sites)	Electrochemical, Mass-sensitive	Active
Electrical	Conducting polymers (Conjugated polymers that intrinsically conduct electricity)	Electrochemical, Electrical	Active
	Electroactive polymers (Polymers that alter structures and/or other properties in the presence of an electric field)		Active
	Highly charged polymers		Active
Thermal	Thermoresponsive polymers (Polymers that undergo structural and/or other changes under heat)	Optical, Electrochemical, Electrical	Passive
Optical	Optically transparent polymers (typically non-absorbent in the visible wavelength range)	Optical	Active
	Photoactive polymers (Polymers that chemical reactions under the exposure of light, typically UV)	Electrochemical	Active
Mechanical	Elastomers (Viscoelastic polymers conforming to the surface in contact)	Optical, Electrochemical, Electrical, Mass-sensitive	Passive
Biological	Biocompatible polymers (Polymers showing no toxicity or other deleterious effect on biological function)	Optical, Electrochemical, Electrical, Mass-sensitive, Magnetic	Passive
